# Bifurcation behaviour of resonant magnetic perturbation control of edge localized modes in tokamaks: nonlinear simulation results

**DOI:** 10.1098/rsta.2022.0161

**Published:** 2023-02-20

**Authors:** Anantanarayanan Thyagaraja, Abhijit Sen, Debasis Chandra

**Affiliations:** ^1^ Astrophysics Group, Bristol University, Bristol BS8 1TL, UK; ^2^ Institute for Plasma Research, Gandhinagar 382428, Gujarat,India; ^3^ Homi Bhabha National Institute, Training School Complex, Anushaktinagar 400094, Mumbai, India

**Keywords:** Type I ELMs, bifurcation, mitigation, suppression

## Abstract

In this article, some novel results of two fluid nonlinear simulations on the control of edge localized modes (ELMs) in tokamaks by resonant magnetic perturbations (RMPs) are presented. Many experiments around the world have demonstrated that RMPs are effective in possibly mitigating or even completely suppressing strong (type I) ELMs that would seriously degrade confinement and could cause other heat-flux problems in both present (e.g. JET) and planned future tokamaks (ITER). Our simulations demonstrate that non-axisymmetric RMPs with toroidal mode numbers n=2,3,4 and suitable field-strengths (kAturns) at the plasma wall imply significant bifurcations in their ability to mitigate or even suppress type-I ELMs, qualitatively similar to RMP effects on ELMs reported in experiments.

This article is part of a discussion meeting issue ‘H-mode transition and pedestal studies in fusion plasmas’.

## 1. Introduction

Edge localized modes (ELMs) feature prominently in high confinement mode (H-mode) operation in advanced tokamak devices. They can expel large amounts of the plasma stored energy and particles with resultant high fluxes of heat that could damage plasma facing components and divertor plates. For future devices like ITER, their control and mitigation/suppression without compromising H-mode confinement is a serious issue. ELMs are known to be caused by sufficiently large current and pressure gradients in typical H-mode edge regions, giving rise to peeling-ballooning mode (PBM) instabilities of a magneto-hydrodynamic (MHD) origin. They have been studied in many theoretical and experimental works [1–6].

The nonlinear dynamics of ELMs in realistic tokamak conditions is very complex and is not yet fully understood in spite of nonlinear simulations by several authors using MHD and two-fluid codes [7–9]. Issues relating to amplitudes, repetition rates and mode spectra of ELMs remain to be elucidated, at least partly due to numerical limitations of codes operating with realistic conditions. Recent works [10–16] have tackled the challenge of explaining the mechanism of resonant magnetic perturbation (RMP) control (by mitigation/suppression) of type-I ELMs observed in several experiments [17–21]. The simulations presented in this article were carried out using the Culham Transporter of Ions and Electrons (CUTIE) [8,12,22–25] – a two-fluid electromagnetic nonlinear global code. The full details related to ELM simulations without and with RMPs are available in [8,12]. In brief, CUTIE evolves the full set of two fluid equations at a scale intermediate between the device size and the ion gyro-radius. A periodic cylinder model of a large aspect ratio tokamak geometry is adopted (*op.cit.* for details and the following section). It was shown in Refs. [8,12] that ELMs are two-fluid versions of the PBMs of MHD that occur due to the pressure gradients created by a particle source in the plasma and a simulated scrape off layer (SOL) using a sink exterior to the plasma. CUTIE is capable of simulating multiple ELM relaxation periods in COMPASS-D [8], as well as ELM dynamics in the presence of RMPs [24,25]. In this article, we aim to address some important bifurcation phenomena observed in recent experiments with RMP control of ELMs in tokamaks. This has been done using the CUTIE code to investigate the character of the nonlinear two fluid, electromagnetic global dynamics of ELMing plasma subjected to RMPs with different toroidal mode numbers n and strengths. The principal objective of such an investigation is to characterize the bifurcations that arise and their effects on the temporal waveforms in the edge region of the turbulence and the ELMs, as well as on the power spectra and resultant transport effects. The main conclusions from our present CUTIE simulations are as follows: (i) there are bifurcation events similar to the experimental observations of ELM mitigation/suppression under well-defined specifications of the RMPs acting on the ELMs and the turbulence, starting with the final states obtained in the two earlier references [8,12]; (ii) it is also found that the strength of the RMP field required for significant reduction of ELMs varies with the toroidal number n of the RMP field and is a minimum for n=3; (iii) as the strength of the RMP is increased beyond the bifurcation point, the post-bifurcation behaviour differs for different n values. For n=2 and 3, the system continues to remain in an ELM mitigated state, whereas for n=4, the system goes back to the initial ELMy state. This is demonstrated by presenting the waveforms and power spectra of the plasma turbulence and ELMs; and (iv) there are significant RMP effects on the profiles and confinement, as already found in [12] for the case of n=2.

In §2, we present a brief description of the CUTIE model including the specifications of the RMPs as regards their modal content and fields applied at the plasma wall. Section 3 presents the details of the results obtained including effects on the waveforms, power spectra and the bifurcation phenomena revealed by the simulations, as the toroidal number n and the strength parameter $K^{*}$ of the RMPs considered are varied over a range of values. The consequences of these bifurcations for ELM control, spectral changes to the drift Alfvénic turbulence spectra and the global confinement are discussed. Section 4 contains a discussion of three possible mechanisms that are possibly implicated in our bifurcation/transition results. We also compare and contrast the results and methodology of our simulations with earlier approaches [10,13,15,26] to the same phenomena. This article ends with a list of caveats attached to our simulations and our principal conclusions.

## 2. Description of the CUTIE model

The CUTIE code as described in details in Refs. [8,12] is based on the following equations of evolution for the electron density n, electron and ion pressures $p_{e,i}=nT_{i,e}$, plasma velocity v and the magnetic field B:

2.1 $$\frac{\partial n}{\partial t}+\nabla \cdot (n\text{v})=S_{p},$$

2.2 $$m_{i}n\frac{d\text{v}}{dt}=-\nabla p+\frac{\text{j}\times \text{B}}{c}+{\text{F}}_{\mathrm{eff}},$$

2.3 $$\frac{3}{2}\frac{dp_{i,e}}{dt}+p_{i,e}\nabla \cdot {\text{v}}_{\text{i, e}}=-\nabla \cdot {\text{q}}_{\text{i, e}}+P_{i,e},$$

2.4 $$\text{E}+\frac{{\text{v}}_{\text{e}}\times \text{B}}{c}=-\frac{\nabla p_{e}}{en}+{\text{R}}_{\text{e}}$$

2.5 $$\;\mathrm{and}\;\nabla \times \text{B}=\frac{4\pi \text{j}}{c},$$

where $n=n_{e}∼n_{i}$, $j=e(n_{i}v_{i}-n_{e}v_{e})$, $p=p_{i}+p_{e}=n(T_{e}+T_{i})$, $v=(m_{i}v_{i}+m_{e}v_{e})/\left(m_{i}+m_{e}\right)∼v_{i}$,$B=\nabla \psi \times b_{\mathrm{tor}}+B_{0}b_{\mathrm{tor}}$ and the electric field, $E=-(1/c)(\partial \psi /\partial t)b_{\mathrm{tor}}-\nabla \phi$ with $b_{\mathrm{tor}}$ being the unit vector in the ‘toroidal’ direction of the equilibrium magnetic field. $S_{p}$ is the external particle source (cf. [8] Section 3), $F_{\mathrm{eff}}$ is the effective force on the plasma, $P_{i,e}$ are the input power sources, $q_{i,e}$ are the heat flux vectors and $R_{e}$ is the electron-ion friction force. Gaussian units are used (some parameters and calculated quantities are expressed in SI for ease of reading). The physics assumptions, constitutive relations, sources and sinks, numerics used and the boundary conditions have been described in detail in the references cited.

The cylindrical coordinates $r,\theta ,\zeta =z/R_{0}$ are employed. The tokamak minor radius is taken to be a and the major radius $R_{0}$. The poloidal angle is θ, while the ‘toroidal’ periodicity coordinate is ζ. The wall boundary is located at r/a=1, and the magnetic axis is at r/a=0. The plasma ‘edge’ is at r/a=0.95, and the region beyond is a representation of the SOL. The poloidal flux function is ψ, and the electrostatic potential is ϕ. All plasma quantities are functions of r,θ,ζ,t. The simulations used the following *fixed* parameters: $R_{0}=0.56\;m$, a=0.24 m, $B_{0}=2.07\;T$, $I_{p}=242\;\mathrm{kA}$, $m_{D}=3.35\times 10^{-27}\;\mathrm{kg}$, time-step $\Delta t=5\times 10^{-8}\;s$, ${\bar{n}}_{0}≃5\times 10^{19}\;m^{19}$ during H-modes. $P_{\mathrm{ech}}=0.5\;\mathrm{MW}$, $P_{\mathrm{ohm}}≃0.75\;\mathrm{kW}$. The radial mesh was 101 with 64 poloidal and 32 toroidal harmonics in the Fourier representations of θ,ζ. The profiles without RMPs are shown in quasi-stationary ELMing periods in figs 6 and 7 of [8], as well as the turbulence in figs 8 and 9. Information about the effects of n=2 RMPs of a predetermined strength are illustrated in [12]. It should be noted that the simulation in that work was a ‘warm start continuation’ from the final state of all variables and parameters at 263 ms from the simulations of [8], apart from a switch-on of the n=2 RMP, as detailed therein. Thus, we can seamlessly continue from previously calculated fields and turn on (or off) any source/equations and investigate the effects on the evolution; there is no further need for any ‘initial’ data. In the present work, the influence of RMPs with n and various field strengths is explored in some detail with a view to explaining several phenomena, including bifurcations observed in recent RMP experiments [13,21]. In the absence of an RMP, the fluctuating part of ψ(1,θ,ζ,t)=0. This is simply the ‘wall boundary condition’ that requires all non-zero Fourier components of ψ(1,θ,ζ,t) to vanish. When an RMP is present, we impose at r/a=1 a $\psi_{\mathrm{rmp}}$ according to the formula, where $K=K^{*}\times 10^{-3}$ is an amplitude parameter:

2.6 $$\psi_{\mathrm{rmp}}(a,\theta ,\zeta )=\left(K\frac{aB_{\mathrm{pol}}}{B_{0}}\right)\sum_{m\neq 0}\left[\frac{1}{m^{2}+1}\right]\cos(m\theta +n\zeta ).$$

We considered n from 1 to 6 for a range of values of the parameter $K^{*}$. From the definitions, it can be verified that $\psi_{\mathrm{rmp}}$ has dimensions of length (when $K^{*}$ is a purely numerical parameter), and since the fluctuating fields $\delta B/B_{0}$ are scaled by $B_{0}$ (the ‘toroidal’ field at the magnetic axis), the fluctuating poloidal flux function has dimensions of length. Hence, the boundary value $\psi_{\mathrm{rmp}}$ yields the radial RMP field perturbation (at r/a=1) upon differentiating with θ, the poloidal angle. Given structure of the RMP coils, K and $K^{*}$ (for any specified n) can be calculated by electrodynamics. In EAST [21], the authors quote 16kAturns in their RMP coils that lead to a few Gauss (i.e. $\delta B_{r}(\mathrm{rmp})/B_{0}≃10^{-4}$). The size of $K^{*}$ thus directly correlates with the radial RMP magnetic perturbation at the wall. In [25], an RMP with n=2, $K^{*}=4$; $K=4\times 10^{-3}$ was assumed.

## 3. Results of simulations

**Figure 1.**
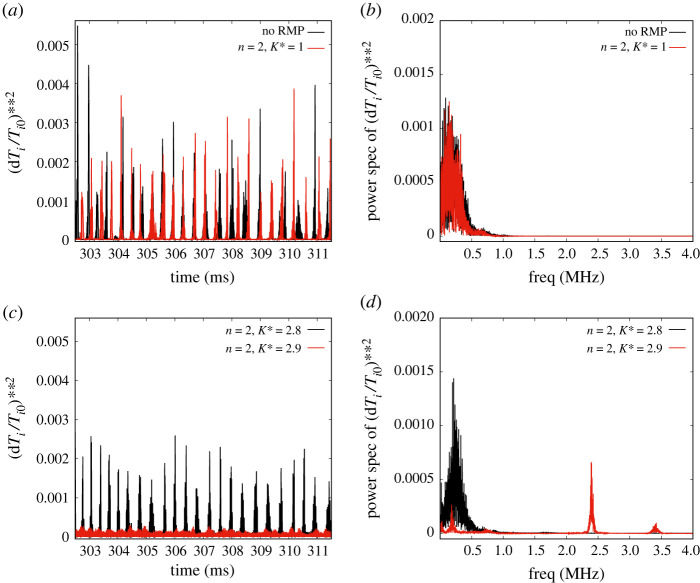
Waveforms of ${\left(dT_{i}/T_{i}\right)}^{2}$ , depicting ELMs, for (*a*) n=2; $K^{*}=0,1$. It shows a slight decrease in the ELM amplitudes due to the application of an RMP of strength $K^{*}=1$. The corresponding power spectrum displayed in (*b*) shows a slight shift to higher frequencies in the presence of RMP. The waveforms in (*c*) for n=2; $K^{*}=2.8,2.9$ show a dramatic drop in their amplitudes for a slight change in $K^{*}$ from 2.8 to 2.9, indicating a ‘bifurcation’ behaviour. The corresponding power spectrum (*d*) also reveals drastic changes at $K^{*}=2.9$ with the appearance of sharp peaks at 2.4 and 3.4 MHz. (Online version in colour.)

We start with a reference calculation that was used in earlier works [25] with no RMPs, showing an ELMing discharge under COMPASS-D conditions, and keeping all physical and numerical parameters fixed. Using this as a ‘control’, and the RMP specification equation (2.6), CUTIE simulations have been made with a set of mode number and strength parameters, $n,K^{*}$. The results of the power spectra are presented, and the waveforms of the turbulence are represented by:

3.1 $${\left(\frac{dT_{i}}{T_{i0}}\right)}^{2}\equiv \Sigma_{m,n}\left[\frac{|\delta T_{mn}^{i}(r/a,t){|}^{2}}{T_{i0}^{2}(0,t)}\right],$$

at r/a=0.9 at time t and $\delta T_{mn}^{i}(r/a,t)$ is the m,n Fourier coefficient of the ion temperature fluctuation [25]. The power spectra are from [0–4] MHz. It should be noted that the ELM “rise-time” is of order 4 μs, corresponding to 250 kHz. Most of the low-frequency drift-Alfvén turbulence is mainly in the 0–250 kHz range. The waveforms in figure 1*a* show that an RMP with n=2, $K^{*}=1$ is only slightly better than the reference ($K^{*}=0$) case. The power spectra in figure 1*b* do show that there is a shift to higher frequencies when the RMP is applied. There is almost no power beyond 1 MHz in both cases. The narrow, tall spikes represent type-I ELMs (as shown in [8,12]). Next we show a remarkable case of a ‘bifurcation’ or transition of the system induced by an RMP with n=2: figure 1*c*,*d* show the waveforms and power spectra for two close values of $K^{*}=2.8\;\mathrm{and}\;2.9$. It is noteworthy that the $K^{*}=2.8$, ‘pre-transition’ waveform has ELMs of somewhat smaller amplitudes than those in figure 1*a* and are also somewhat more regular. Its power spectrum figure 1*d* has almost a Gaussian structure, with a sharper peak at 250 kHz. However, when $K^{*}=2.9$, the waveform changes drastically: we find a grassy, low amplitude ($10^{-3}$) background with some gentle peaks at roughly the same frequency as the ELMs seen ‘pre-transition’. In figure 1*d*, we see the same drastic change reflected in the power spectrum which acquires sharp peaks at 250 kHz, a stronger, even narrower ‘line spectrum’ at about 2.4 MHz and a much smaller one at 3.4 MHz. At $K^{*}=2.9$, we observe that it is not only the low-frequency spectrum, but the higher frequency ‘grassy’ parts of the turbulence are also affected by the bifurcation.

**Figure 2.**
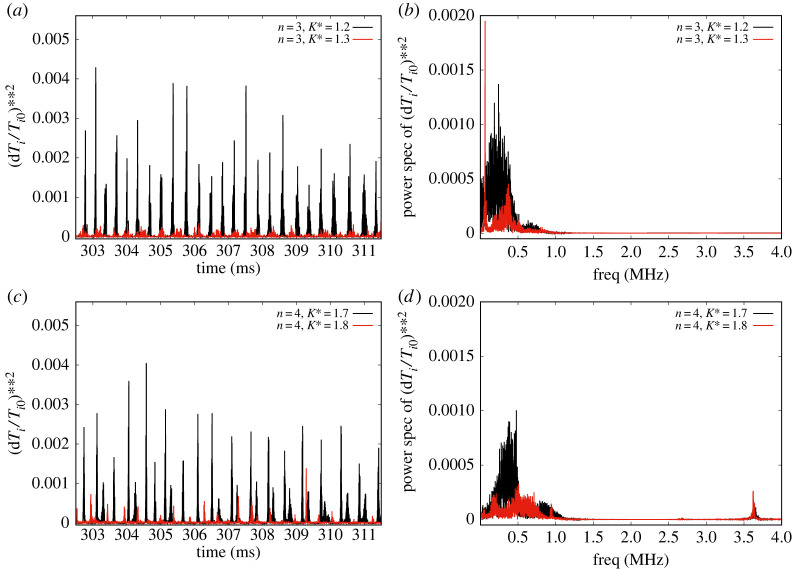
Depiction of waveforms of ELMs for (*a*) n=3, $K^{*}=1.2,1.3$ and (*c*) n=4, $K^{*}=1.7,1.8$ showing bifurcation behaviour for these higher values of n. Their corresponding power spectra are shown in (*b*) and (*d*), respectively, and exhibit similar post-transition characteristics of sharp high-frequency peaks. (Online version in colour.)

The transitional case for an RMP with n=3 is considered in figure 2*a*,*b*. We find that the transition is rather dramatic and occurs at the relatively low value of $K^{*}=1.25$, as compared with the preceding case. We observe that in figure 2*a*, the waveform amplitudes are actually somewhat larger than the corresponding one for $n=2,K^{*}=2.8$ in figure 1*c*. The post-transition waveform in figure 2*a* for $n=3,K^{*}=1.2$ consists of a very low ‘oscillatory’ grassy broadband turbulence with small peaks occurring at roughly the ELM repetition frequency, pre-transition. The post-transitional power spectrum in figure 2*b* is dramatically different from that in the $n=2,K^{*}=2.9$ case: it exhibits a very low-frequency (around 50 kHz) line-spectral peak (a coherent mode?) at high amplitude and a Gaussian, relatively broad profile continuous spectrum centred around 400 kHz. There are no other peaks and no power beyond 1 MHz, totally unlike figure 1*c* post-transitional spectrum.

For completeness, RMPs with n=4 exhibiting a turbulence transition are shown in figure 2*c*,*d.* The transition occurs at $K^{*}=1.7-1.8$. The pre-transition waveform figure 2*c* is somewhat irregular and apparently with sharp type-I ELMs and almost no broadband turbulence (apart from broadening of some ELMs). The transition leads to much smaller, ‘mitigated’ ELMs with some small amplitude broadband fluctuations, as shown in figure 2*c*. The pre-transition power spectrum in figure 2*d* is already significantly lower in amplitude in comparison with n=2,3 cases at pre-transition. It exhibits a relatively broad peak around 350 kHz and a line feature close to 500 kHz. Moreover, there is a broad smaller peak around 800 kHz that is unlike the n=2 but resembles qualitatively the n=3 pre-transition spectrum. Post-transition, n=4, $K^{*}=1.8$ exhibits some similarities to the n=2 and the n=3 cases: firstly, the overall amplitude level seems to be lower. There are three spectral peaks below 1 MHz and a flat broadband feature from 500 to 750 kHz. Then, unlike n=2, n=3, there is a relatively sharp Gaussian spectrum around 3.6 MHz. We have carried out simulations with n up to and including n=6, but do not present them here due to a lack of space.

**Figure 3.**
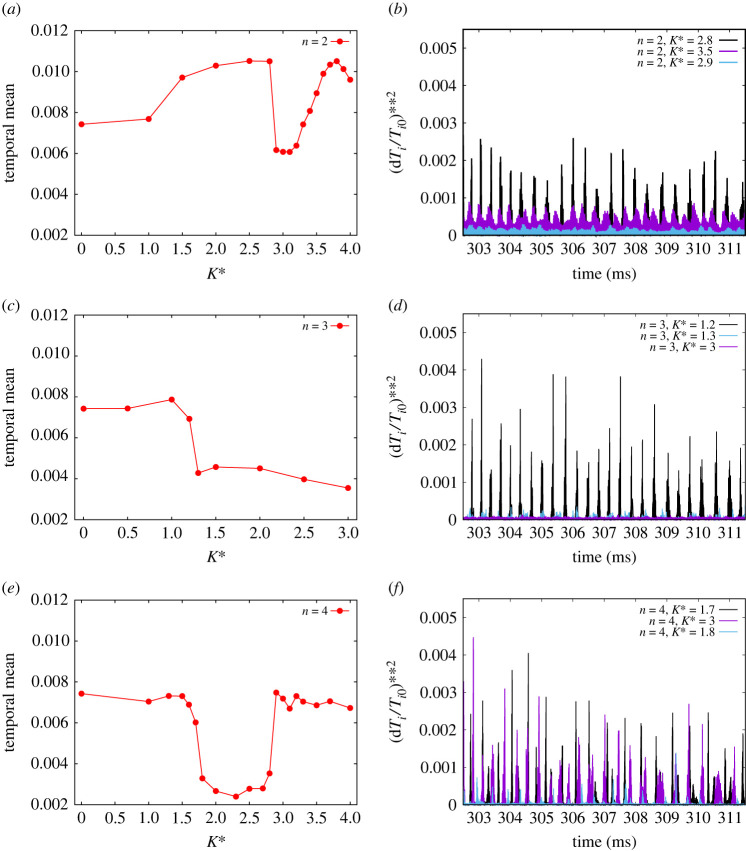
(*a*–*f*) A comparative view of the temporal means and waveforms of ELMs as a function of $K^{*}$ for n=2,3 and 4. It is to be noted that the bifurcation behaviour of the n=3 is distinctly different from the n=2 and 4 cases. While the temporal means for n=2,4 rises up sharply after the drop at the bifurcation point, that of the n=3 continues to go down in a broad manner. There is also a subtle difference in the post-bifurcation nature of the ELMs for the n=2 and n=4 cases as evident from the waveforms. While the n=2 post-bifurcation waveforms remain mitigated, those for the n=4 case revert back almost to their original unmitigated state. (Online version in colour.)

**Figure 4.**
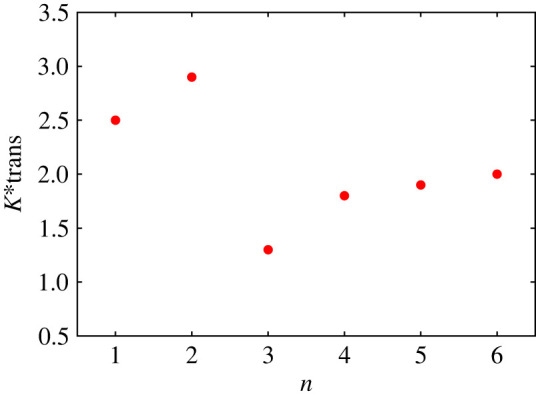
A consolidated plot showing the variation of the threshold value of $K^{*}$ for bifurcation to occur as a function of the toroidal mode number n of the RMP. The curve shows a distinct minimum for n=3, suggesting an optimum mode number for effective implementation of RMP mitigation of ELMs. This is one of the principal findings of the present study. (Online version in colour.)

The fact that the waveforms and spectra of the system subjected to an RMP with *fixed* n as $K^{*}$ are varied undergo a bifurcation-like change in an otherwise ‘smooth’ evolution is illustrated in figure 3*a*–*f*. In figure 3*a*, the temporal means (i.e. a time average) of the waveforms for n=2 and each $K^{*}$ chosen are plotted. It shows that, starting with $K^{*}=0$ (i.e. the ‘Reference NO RMP case’), after an initial slow linear increase up to $K^{*}=1$, the values trace (as $K^{*}$ is increased by 0.5 at each step) a rising curve, apparently flattening around $K^{*}=2.5$. Then, at $K^{*}=2.8$, we see a sudden drop of the temporal mean to a significantly lower value at $K^{*}=2.9$. This then is the ‘transition’ experienced by the system. As we continue increasing $K^{*}$, there is a smooth but small drop to a minimum (around $K^{*}≃3$) followed by a relatively smooth but steeper rise to a peak around $K^{*}=3.5$, where the time mean is almost back to its earlier value at 2.8. In figure 3*c*, the corresponding time mean results obtained by CUTIE are shown for the n=3 RMPs. As noted earlier, the steep transition is shown at 1.2–1.3, as $K^{*}$ is varied. However, unlike n=2, there is a smooth increase to a further flat maximum followed by a smooth decrease to a lower final value at $K^{*}=3$. We next show that for n=1,2,3,5.0,6, starting with $K^{*}=0$, the bifurcation occurs at the corresponding transitional value of $K_{\mathrm{trans}}^{*}$. In figure 4, the ‘first critical values’ of $K_{\mathrm{trans}}^{*}$ are plotted. Several interesting features emerge from this ‘transition plot’:

(1) $K_{\mathrm{trans}}^{*}(n=1)=2.5<K_{\mathrm{trans}}^{*}(n=2)$.
(2) $K_{\mathrm{trans}}^{*}(n=3)≃1.25$ is the minimum transitional value.
(3) For larger n, the values increase smoothly and appear to ‘saturate’ at around n=6, the largest value simulated. It is also evident from figure 4 that n=3 is ‘optimal’ for the RMP configuration in terms of the lowest transitional $K^{*}$.

In principle, therefore, our two-fluid CUTIE model predicts both the transition, the near-complete mitigation of the type-I ELMs and the existence of a ‘point/discrete spectrum (coherent modes)’ after transitions, which may manifest as observable oscillations near r/a=0.9, near the plasma edge. These findings bear a close qualitative resemblance to recent experimental observations [21] and may have important implications for RMP suppression of ELMs on ITER [27]. We next turn to the question of how the transitions in the turbulence (both waveform amplitudes and spectral characteristics) affect the global properties such as ‘Density pump out’ and β(%)) post-transition. In [12], the effects of switching on an RMP ($n=2,K^{*}=4$) were considered on $\beta ,\tau_{E},\tau_{p}$ with the density feedback: it was shown that while, after an initial transient, β, $\tau_{E}$ increase, the particle confinement is slightly degraded relative to the no RMP case, in spite of the feedback control of the plasma particle number, $N_{\mathrm{tot}}(t)$; this occurs under the assumed conditions of the switch-on of a constant RMP field. The experiments show that both particle and energy confinement can be degraded after an RMP is used, especially when its strength is increased in time. The modelling of the SOL used in CUTIE is possibly overly simplified. It is also possible that while the ELMs are mitigated, the grassy broadband actually increases or the energy source profiles in experiment change due to the changes observed in the plasma profiles in experiments. In this article, our focus is not on the density pump out but on the effects of RMPs on β.

**Figure 5.**
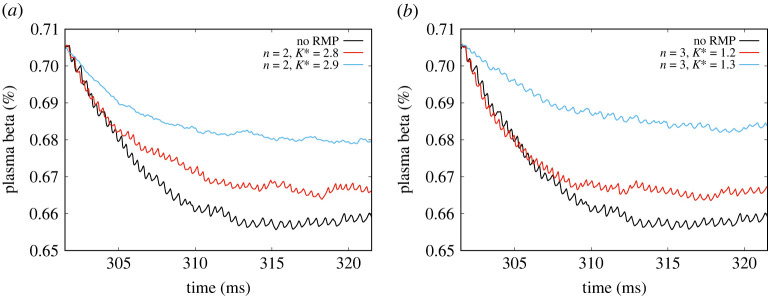
Temporal evolution plots of β(t)(%) close to the bifurcation points for (*a*) n=2 and (*b*) n=3. It is seen in both cases that the pre-bifurcation (‘sub-critical’) values of β are only marginally higher than the no-RMP cases. However, the post-bifurcation (‘super-critical’) values are substantially higher than the no-RMP case. (Online version in colour.)

In figure 5*a*, β(t) (%) of the no-RMP case is plotted along with those for the n=2, $K^{*}=2.8$ ‘sub-critical’ RMP and the corresponding n=2, $K^{*}=2.9$ ‘super-critical’ RMP. It can be seen that the sub-critical case is not significantly higher than the no-RMP reference case after 16 ms. However, the super-critical n=2, $K^{*}=2.9$ curve reaches a saturated β that is 2% higher. Figure 5*b* shows that with n=3, the sub-critical value itself is a little larger than the reference value. The super-critical value is significantly higher than the sub-critical one, almost by 3%. A similar trend, not shown, is observed for n=3,4.

It is important to bear in mind that the plasma thermal energy that essentially controls β varies relatively slowly, and the values quoted may not be the ones appropriate for a true long time-scale estimate of any improvement due to the turbulence. However, it seems that the decrease of the turbulence after the transition does affect the edge heat transfer to the region between the plasma edge r/a=0.9 and the device wall at r/a=1.

## 4. Discussion and conclusions

The purpose of this work is to advance the ideas and results we had obtained in [8] on ELMing plasmas and in [12] on how n=2 RMPs with given strengths influenced the two-fluid plasma dynamics of the ELMs and turbulence. There are four essential ingredients to the proposed model: (1) the edge instabilities are driven by current and pressure gradients that form in H-modes, namely, the PBMs modified by two-fluid drift Alfvénic physics; (2) these modes must be allowed to evolve nonlinearly and thereby interact with both the m=n=0 ‘profiles’ but also all the turbulence in the edge-pedestal region and modify both and saturate and decay due to the significant profile changes due to the resulting enhanced fluxes to the SOL; (3) the combination of a particle source within the plasma and the rapid clearance of plasma via the divertor and the resultant low density in the SOL is a necessary condition to produce the radial gradients required to be generated for the ELM relaxation oscillations; and (4) the RMPs themselves are modelled in terms of the radial magnetic perturbations they impose on the plasma through equation (2.6). In [26], eqn (7), a simpler version of a similar boundary condition, was employed to represent the RMP field.

There is no simple mathematical/physical model available at present to explain the phase transition-like behaviour seen in our complicated, three-dimensional electrodynamical nonlinear system. We identify three possible mechanisms that can, individually or in concert, account for the bifurcation behaviours found in the CUTIE model resembling qualitatively observations in many recent experiments.

The first mechanism involves a direct cascade from the relatively long wavelength ELMs (an ‘inertial scale’) to much shorter wavelength micro scales (relatively higher m≃nq(r/a)), where neoclassical and turbulent diffusivities in the code can dissipate (and diffuse) the energy to saturate the mode by both reducing the gradients (observed experimentally) and driving up the stable, non-resonant modes via linear *and* nonlinear mode coupling. This process is clearly present even without RMPs. When the RMPs are present with helical structure $n_{\mathrm{rmp}}\geq 2$, and their strength is sufficiently large to interact with the rotating PBMs as well as many other non-resonant modes in the spectrum, the latter dump their magnetic and kinetic energies in the grassy background and thus tend to quench the ELMs.

The second mechanism is related to the first, since $n_{\mathrm{rmp}}$ could produce a resonant plasma mode with $m≃q(r/a)n_{\mathrm{rmp}}$. For q(r/a=0.9)≃4, relatively high m sidebands can be excited. In [12], we have discussed these interactions and present evidence for spectral changes relative to the ‘no RMP’ case in the radial and Fourier domains. Although that study investigated $n_{\mathrm{rmp}}=2$, the same phenomena are expected for all other values of $n_{\mathrm{rmp}}$. Our simulations do not consider externally driven plasma flows (essentially in the ion fluid). However, the electron fluid possesses a very considerable sheared flow thanks to the large parallel current density as well as substantial perpendicular transverse flows as implied by the two-fluid electron momentum balance equation, equation (2.4), illustrated by the zonal flow evolution during ELMing in [8]. It is well known from fluid dynamics that this vorticity can lead to two effects: it can cause ‘phase mixing’ and damp the turbulence in the excited small scales. It can also destroy the linear eigenfunctions of the PBMs by greatly reducing their radially coherent structure. While this can lead to saturation of ELMs, it is not clear whether the presence of RMPs of high enough strength can enhance this process to result in ELM mitigation, nor why it should only occur at certain ‘eigenvalues’ of $K_{\mathrm{trans}}^{*}$. It is notable that this mechanism suggests that RMPs not only influence the dynamics of larger scale modes like PBMs and profiles but also the smaller scale drift-Alfvén modes involved in the turbulence.

A third mechanism that could actually explain the bifurcations as the strength $K^{*}$ is varied for a fixed $n_{\mathrm{rmp}}$ will now be described. In the plasma frame, the RMP appears to rotate, a fundamentally two-fluid effect. This rotation rate clearly depends on the position within the plasma and the drift-Alfvén wave characteristics at that location. The system can give rise to modulational instabilities of the type discussed in [28]. This is analogous to applying a time-periodic perturbation to a nonlinear dynamical system with a very large number of degrees of freedom. There is then the possibility of bifurcations of the motion when a given periodic motion loses its stability at a critical point dependent on the interaction strength and frequency of the external perturbation. Therefore, we have a situation analogous to, but far more complicated (due to more complex three-dimensional physics) than encountered in nonlinear dynamics of a nonlinear dissipative system (e.g. a damped simple pendulum or a Van der Pol oscillator) driven by a periodic or quasi-periodic external force [29]. The nature of bounded motions (both regular, periodic or quasi periodic or chaotic, involving strange attractors) already has a voluminous literature and requires very delicate analyses [29] and involves many bifurcations (including entrainment/mode-locking of the system frequencies and phase) as the driving force is increased. Extending such techniques to systems like tokamaks (or even fluid dynamic phenomena of turbulent flow like re-laminarization or the Prandtl drag crisis in flows past bodies) is a formidable challenge that still awaits satisfactory analytical solutions.

At the present time, which (if any) of the suggested mechanisms are responsible for the bifurcations induced by equation (2.6) on ELMs and turbulence found in our CUTIE simulations of RMP effects remains unresolved. The elucidation of this crucial issue needs more extensive investigations and guidance from further experiments such as those reported by the EAST Team [21] and others.

It is useful to briefly compare other recent computational approaches with ours. In [26] (and references therein), the authors employ equations based on two-fluid physics in the their TM-1 model, as does CUTIE. Although TM-1 is a reduced model, analogous to Strauss MHD equations, but incorporating compressiblity, electron thermal conduction, ion momentum equations and a generalized Ohm’s Law in cylindrical geometry, CUTIE treats the full set of two-fluid equations also in cylindrical geometry (taking account of the radial variation of the equilibrium toroidal field in implementing linear mode coupling) including the ion energy equation and solves for the turbulence which determines the transport prevailing in addition to neo-classical transport under specified sources and boundary conditions. This means that transport coefficients are calculated self-consistently including the profile–turbulence interactions that are key to the dynamics of the system evolved by the code. Thus, it is able to evolve the profiles as they respond to the turbulence and the turbulent spatial and temporal spectra. It is this feature that enables RMPs to influence both the PBMs and the turbulent modes. The overall volume-averaged plasma density is ‘feed-back’ controlled to a prescribed value [8]. In particular, the CUTIE equation (2.4) shows that if dissipation and pressure effects are absent, the magnetic field is frozen in the *electron fluid*, a key difference from visco-resistive MHD.

Recent simulations involving the JOREK code [10,13,15] involve modelling the ELM-RMP interaction phenomena observed in ASDEX and KSTAR. JOREK is a three-dimensional nonlinear MHD code in full toroidal geometry, capable of handling X-points and SOL. It uses a five-field reduced MHD model including two-fluid effects. All sources and transport coefficients are assumed to be constants in time, and the equilibrium profiles are obtained from experiment and held fixed while the nonlinear dynamics of the fluctuating fields (essentially PBMs) are calculated. The code works in the quasi-neutral regime (like CUTIE), but also assumes $T_{i}=T_{e}=T$. The code uses fixed Braginskii viscosity and an enhanced resistivity, an order of magnitude larger than Spitzer. It was stated in [10] that the lack of two-fluid modifications of MHD in that version resulted only in a single ELM crash, unlike CUTIE [8], which was a full two-fluid turbulence simulation (not reduced MHD with fixed equilibrium profiles or enhanced resistivity). The authors carefully listed their views about RMP mitigation/suppression of ELMs, which are worth recalling in the light of our ideas stated earlier: in [13], they state that their restriction of mode numbers to n=8 might have prevented a direct cascade to finer scales and possibly inhibited inverse cascades, the most important of which is to the profiles via Smagorinski types of ‘self-consistent’ turbulent diffusivities employed in CUTIE dynamics of profile-turbulent interactions [30]. The authors also state that ‘future nonlinear two-fluid modelling’ would be required to fully account for an enhanced peeling response. Interestingly, they suggest that profile gradient changes due to RMPs is probably not the correct ELM suppression mechanism, rather than the linear toroidal coupling between the ELMs and the RMP-induced mode. This is closely related to the third mechanism we have proposed of the interaction among the ELMs, plasma turbulence and the RMP fields in the presence of relative rotation. They also suggest that more complex interactions exist which need further investigation. In [16], JOREK simulations suggest that the RMPs can nonlinearly saturate the ELMs, resembling somewhat the much reduced spikes seen in our spectra with the repetition rate of the original, unsuppressed ELMs. The authors conclude with suggestions referring to polarization drifts, and turbulence might be important ingredients. CUTIE includes ion inertial effects but not $c/\omega_{pe}$ (electron inertia), as well being a turbulence simulation. Thus, CUTIE and JOREK appear to have complementary strengths and weaknesses.

The important conclusions we can draw from our simulations are the following: (1) starting with the same two-fluid model and results published in [8,12], adding a simplified RMP model with two parameters, $n,K^{*}$, a series of effects on ELMs have been obtained; (2) we show that for n=2,3,4, as the strength parameter $K^{*}$ is increased from zero, the ELMs that are present at $K^{*}=0$ experience a steep transition to an ELM-free state which is clearly exhibited in both the waveforms and power spectra at r/a=0.9; (3) if we plot the transition $K_{\mathrm{trans}}^{*}$ against the $n_{\mathrm{rmp}}$ values, there is a strong minimum for n=3, followed by a smooth increase of the transitional strengths; (4) we have also found evidence of ELM-suppressed cases in which there is a low frequency ‘coherent mode’ of significant amplitude; and (5) preliminary studies of $n_{\mathrm{rmp}}=4$ indicate that after the first transition, substantial increases in $K^{*}$ can actually result in a ‘back transition’. This finding is yet to be investigated and confirmed. CUTIE thus leads to qualitative predictions of RMP effects on type-I ELMs that can be experimentally verified or falsified by performing experiments which use differing n and with constant (in time) strengths, varied over a range of values.

We note that the CUTIE model involves a number of physical assumptions and numerical representations that could conceivably impact upon the RMP-induced edge turbulence/ELM bifurcation results we have presented. The toroidal geometry of tokamaks involves non-circular plasma boundaries; gyro kinetic effects are not included in our two-fluid model. The neglect of electron inertia terms in equation (2.4), the lack of explicit inclusion of SOL physics and the simple modelling of the RMP field at the wall (via equation (2.6)) are some of the idealizations of the model. Earlier work has shown that despite such idealizations, CUTIE simulations do account for ELMs and general effects on them by RMPs, external flows and pellets, at least qualitatively capturing the characteristics. Further work, possibly with higher resolutions including more physics, as well as experimental validation of the predictions of our simulations, will be required to affirm them.

## Data Availability

The reference list in the paper contains all the publications/models we employed from our earlier papers. The figures provide all the information necessary for reviewers and readers. The CUTIE code used itself has long been published in many journals.
